# The use of natural media amendments to produce kale enhanced with functional lipids in controlled environment production system

**DOI:** 10.1038/s41598-018-32866-5

**Published:** 2018-10-03

**Authors:** Natalia P. Vidal, Huong T. Pham, Charles Manful, Ryley Pumphrey, Muhammad Nadeem, Mumtaz Cheema, Lakshman Galagedara, Adedayo Leke-Aladekoba, Lord Abbey, Raymond Thomas

**Affiliations:** 1 School of Science and the Environment/ Boreal Ecosystem Research Initiative, Grenfell Campus, Memorial University of Newfoundland, Corner Brook, A2H 5G4 Canada; 2 Department of Plant, Food, and Environmental Sciences, Dalhousie University, Truro, NS B2N 5E3 Canada; 3 Department of Environmental Sciences, COMSATS University of Islamabad, Vehari, 61100 Pakistan

## Abstract

Diets high in vegetable consumption is highly correlated with reduced risk of developing common lifestyle related diseases. We investigated the effects of three natural growth media amendments [potassium humate, dry vermicast, volcanic minerals or Promix alone (Control)] in enhancing the accumulation of functional lipids in greenhouse grown kale. Functional lipids (n9, n6, n3 fatty acids, diglycerides, galactolipids and phytosterols) were assessed using either gas chromatography/mass spectrometry (GC/MS) or ultra-high performance liquid chromatography-high resolution tandem mass spectrometry (UHPLC-HRMS/MS). The results showed volcanic minerals and dry vermicast were the most successful in enhancing the accumulation of functional lipids in kale. For example, dry vermicast enhanced the accumulation of total C18:1n9 and C16:3n3 fatty acids, while total C18:2n6 fatty acid accumulation was enhanced by volcanic minerals. In conclusion, natural growing medium amendments are remarkably effective in modulating the accumulation of functional lipids in kale grown under controlled-environment conditions. This could be a useful strategy for functional foods production in control environment production systems. Increase access to kale with enhanced functional lipids could aid in increase consumption of these health promotive compounds in the diet with potential implications in population health.

## Introduction

Functional food refers to natural or processed food products in which added or existing ingredients are determined to contain bioactive compounds with known health benefits beyond basic nutritional needs^1^. Functional foods are an emerging field in food science, and widely predicted to become one of the biggest industry trend in the next 25 years^1^. This is driven by increasing demands for functional food products by more health-conscious consumers. As such, many research focuses on the health benefits of existing and new bioactive phytochemicals (functional ingredients) in foods, and the role of a healthy diet for optimal health maintenance and reduction in the risk factors associated with developing common lifestyle related diseases. It is now universally accepted that the health of a population is influenced by diet^2^. Several studies have reported that reduction in the risks for developing lifestyle related illnesses (e.g. diabetes, arthritis, cancer and cardiovascular diseases) are associated with diets high in vegetables, such as kale (*Brassica oleracea* var. acephala D.C)^3–6^. This reduced disease risk is related to high levels of bioactive phytochemicals (functional ingredients) present in the plant tissues^5,7^. Of these bioactive phytochemicals, plant lipids have generated considerable interests as functional ingredients in the development or production of functional foods^4,6^. These include polyunsaturated fatty acids, oleic acids, diglycerides, medium chain triglycerides, galactolipids, phytosterols, fatty alcohols, and carotenoids^4,6,8–10^. The term functional lipids is sometimes used to denote the demonstrated beneficial effects of these bioactive lipids beyond basic nutrition; leading to an improvement in human health or a decrease in disease risk^11^. Polyunsaturated fatty acids (PUFA) include fatty acids with long chains (C_16_ to C_24_), of which C16:3n3, C18:3n3, C18:2n6 predominate in plants^4^. PUFAs including omega 6 (n6) and omega 3 (n3) fatty acids are considered essential fatty acids because the human body is unable to desaturate C_3_ and/or C_6_ in the fatty acid moiety and need to be consumed trough the diet. Consequently, these fatty acids must be obtained from dietary sources^4^. Green leafy vegetables, i.e. kale, are great dietary sources of n3 fatty acids^4,6,12^. In fact, over 54% of kale fatty acids were observed to be n3 fatty acids and over 20% as n6 fatty acids^12^. The literature is replete with documented evidences of the health benefits of n3 PUFA in decreasing the risk of stroke/impaired cognitive function, reduce blood pressure and insulin resistance in humans^11,13,14^. Additionally, the consumption of n3 PUFA has been associated with improvements in the health outcomes of ADHAD (attention–deficit/hyperactivity disorder), schizophrenia, and depression patients^15^. On the other hand, n6 PUFA have been reported to reduce plasma or serum total and LDL (low-density lipoproteins) cholesterol level leading to reductions in these risk factors associated with developing cardiovascular diseases^13^.

Galactolipids also occur in very high concentration in kale and are known to be major sources of esterified oleic (n9), n3 or n6 fatty acids in plants^6^. The fatty acids are esterified at C_1_ and/or C_2_ of the glycerol moiety, while one or two galactose molecules maybe esterified at C_3_ of glycerol to form the head group of the following subclasses of galactolipids: monogalactosylmonoacylglycerides (MGMG), sulfoquinovosyldiacylglycerides (SQDG), monogalactosyldiacylglycerides (MGDG), digalactosylmonoacylglycerides (DGMG) and digalactosyldiacylglycerides (DGDG)^6,9^. Galactolipids are abundant in the chloroplast of plants as such, green leafy vegetables are a superior source^6,9^, and over 90% of the total fatty acids esterified in vegetable MGDG were observed to be n3 PUFA (C18:3n3)^6^. Several studies have demonstrated the bioactivities of galactolipids from vegetables as important nutraceuticals with documented effects on anti-inflammatory diseases and reduced cancer risks^6,16^. This has led to the application and acquisition of several recent patents in relation to the use of galactolipids in the development of plant medicines and functional foods for cancer and anti-inflammatory illness prevention^6,17–20^. Consequently, galactolipids are a class of functional lipids currently used in the development or production of functional foods^6^. Diglycerides (DG) are another new class of functional lipids common in vegetables, however, they exist in low concentration (1–10% w/w) compared to triglycerides^21^.

Structurally, diglycerides are distinct from triglycerides because they contain two fatty acids esterified to a glycerol backbone whereas triglycerides have three. This esterification occurs at either C_1_ and C_2_ or C_1_ and C_3_, leading to the formation of several isoforms of DG with metabolic characteristic distinct from triglycerides; and as such, DG are less likely to be stored as body fat^6,9^. Consequently, DG have been demonstrated in several clinical studies to be effective in suppressing obesity and lowering post prandial serum triglycerides^8,21,22^. Obesity is a major risk factor associated with developing diabetes mellitus, heart disease, some type of cancers and hypertension^21,23^. Considering dietary DG suppress obesity, it also reduces the risks of developing these common lifestyle related illnesses. Currently, DG content is enhanced as a functional ingredient in many edible oils (functional oils) and functional food products to capitalize on these health benefits. In many of these scenarios, DG are mixed with phytosterols as the bioactive ingredients used to develop the targeted functional foods with the potential to combat obesity, metabolic syndrome and diabetes^8,24^. Phytosterols have also attracted great attention as a functional lipid, because scientific evidences have demonstrated their effectiveness in lowering plasma low density lipoproteins and cholesterol levels, which are a known risk component related to cardiovascular illnesses development^8^. However, as in DG, phytosterols are common in vegetables including kale, but they exist in low amounts^8^.

The concept of functional food development or production is aimed at increasing the contents of beneficial functional ingredients (e.g. functional lipids) in the diet, so they are more available and accessible to the consumer. This is of relevance considering the increase cost of health care, an aging population, and the appreciation that lifestyle habits along with dietary patterns constitute modifiable risk factors related to coronary diseases, cancer, type 2 diabetes, obesity and osteoporosis^2,11,25^. Modulation of the growth conditions to selectively enhance the levels of targeted functional ingredients in selected crop varieties cultivated under controlled environmental conditions is an approach for the development of functional foods. Green leafy vegetables, i.e. kale, are of high interest among crop producers as targets for functional foods development, particularly due to superior phytochemical profile, and strong relationships between increase consumption of these vegetables and reduction in the risks of developing common lifestyle related diseases^7,12,26^. Kale is a vegetable crop highly desired by consumers for its versatility in multiple cuisine, ease of cultivation and perceived health benefits^12,26^. The perceived health benefits are associated in part to superior omega 3 levels in kale, which is among the highest in vegetable plants. It is also an excellent source of other bioactive functional lipids, and the lipid content can vary significantly with cultivar and crop growth conditions^12,26,27^.

Plant lipids are known to be important sensors for perceiving and responding to the growth media nutrient composition and climatic conditions. For example, the MGDG/DGDG ratio was observed to be very stable when plants were grown under favorable nutrient regime and controlled-environment conditions. However, when this steady state was altered by modulating the nutrient composition of the growing medium, the response of the lipid content of the crop was significant^27,28^. Natural media amendments, including vermicompost, volcanic minerals and humates, are of considerable interest in the production of vegetable crops under controlled environmental conditions^29^. They are made of non-humic and humic compounds, providing plants with phytohormones, nutrients, free amino acids, humins, fulvic or humic acids; and are important in the growth and development of the plant^30^. Some studies indicated that natural growing media amendments are superior compared to synthetic chemical fertilizers, particularly when used in controlled environment production systems^29,30^. The superiority (advantage) of natural media amendments over synthetic fertilizers include low technology, higher availability and diversity of nutrients, increased beneficial microbial activities, low cost and a reduction in the environmental pollution risks^31^. The chemical composition is known to vary or differ between vermicompost, volcanic minerals, and potassium humates^29^. This variation could modulate the accumulation of functional lipids and other phytochemicals in the cultivated crops. Taking these into consideration, we hypothesize that natural plant growth media amendments could be used under controlled environmental conditions to produce kale plants enhanced with functional lipids, and that this could be a potential production system suitable for the cultivation of kale as functional food. This study was designed to test this hypothesis.

## Results and Discussion

### Polyunsaturated fatty acids (PUFA) as functional lipids in kale

**Figure 1:**
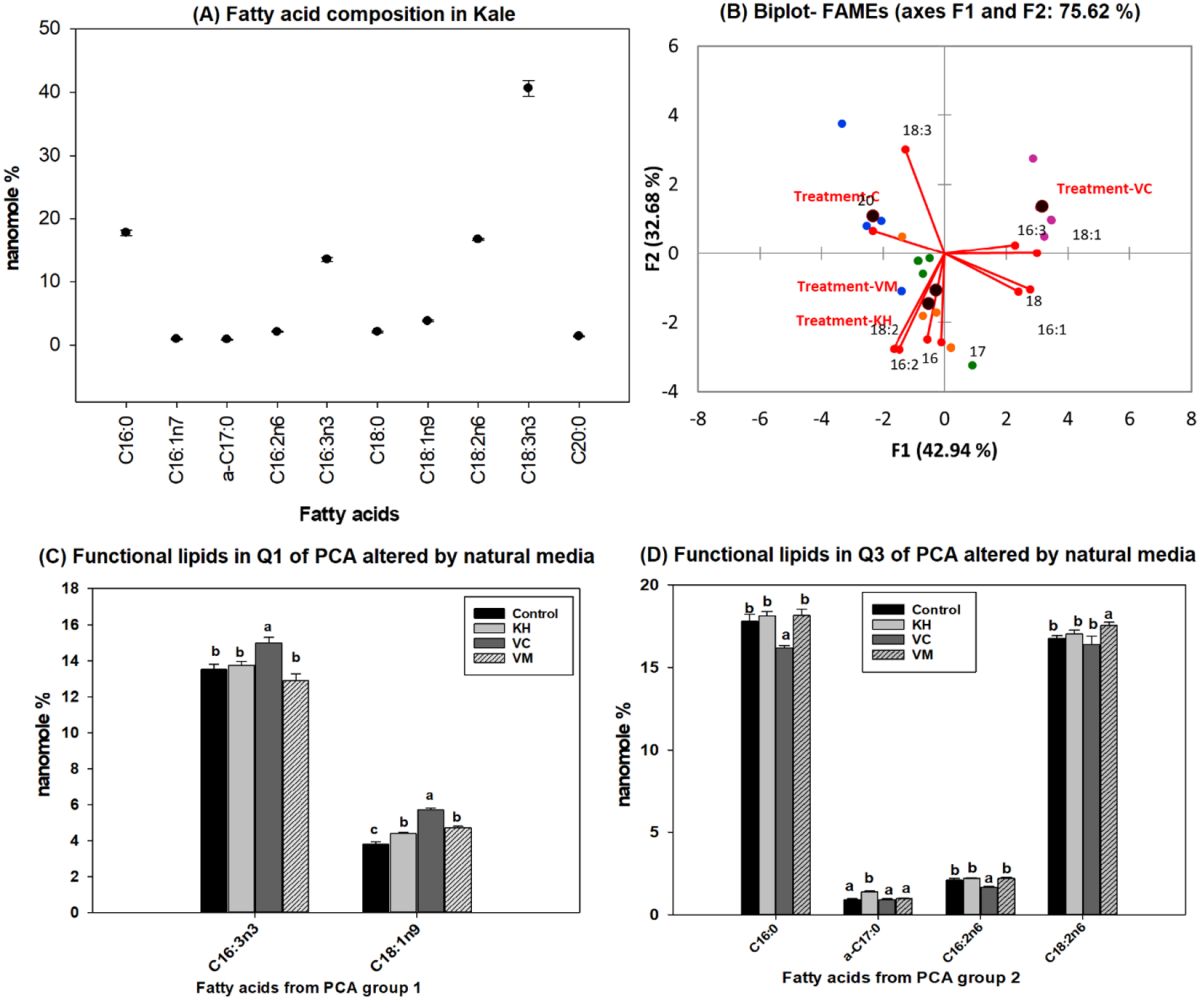
(**A**–**D**). Natural amended media alter the total fatty acid composition of Kale cultivated under control environmental (greenhouse) conditions. (**A**) Observed kale fatty acids, (**B**) Biplot showing relationships between observed kale fatty acid levels and media amendments used for growth. One-way ANOVA showing altered Kale functional lipids segregated in (**C**) quadrant 1 (Q1) and (**D**) Q3 of correlation circle or biplot following principal component analysis. Values in bar chart (nanomole percent by weight composition) represent means ± standard errors. Means in the same row accompanied by different superscripts are significantly different at LSD α = 0.05, n = 4 per experimental replicate. Control = no media amendment added, KH = potassium humate, VC = dry vermicast, VM = volcanic minerals amendments added to the control media. Please note Fig. **A** in graphs of kale lipids reported in this paper represent the composition observed in the control plants.

Mono and polyunsaturated fatty acids, diglycerides, galactolipids and phytosterols were the targeted functional lipids evaluated in this study. Analysis of the total kale fatty acids as methyl esters revealed that C16:2n6, C16:3n3, C18:1n9, C18:2n6 and C18:3n3 were the major functional fatty acids present in kale regardless of the type of natural media amendment used for cultivation (Fig. 1A–D). PUFA (n3 and n6) are essential fatty acids because the body cannot desaturate fatty acids at C_3_ and C_6_ positions, counting from the carboxylic acid moiety. As such, these must be included in the diet. The results of this study showed kale is a good dietary source of n3 fatty acids because over 54% of the total kale fatty acid composition is n3 fatty acids. Approximately 20% were n6 fatty acids (Fig. 1A–D). The composition observed is consistent with previous findings reported in the literature^4,6,12^. The literature is replete with documented evidences that demonstrate the effectiveness of functional lipids in modulating human physiology and reducing the susceptibility to heart diseases, obesity, depression, atopic dermatitis, Alzheimer’s and Parkinson’s disease^15,23^.

All the natural amended media evaluated in this study were observed to be effective in modulating the level of functional lipids present in kale (Fig. 1B–D). The biggest overall effect of the natural media amendments appears to be on the delta 9 desaturase enzyme that catalyzes the desaturation of the C18:0 into C18:1 fatty acid. This is because all three natural media amendments enhanced the levels of oleic acid (C18:1n9) in kale (Fig. 1C). However, dry vermicast amended media was significantly (p < 0.01) the most effective of the media amendments in enhancing the level of C18:1n9 in kale. Consumption of foods high in oleic acids (C18:1n9) has been associated with reduced risks of developing cardiovascular diseases^11,23^. This is particularly evident in Mediterranean diets, which contain high levels of oleic acids^32^. In addition to oleic acid, an accumulation of n3 fatty acids was observed in kale cultivated in dry vermicast media. This enhancement was observed only in the level of C16:3n3 (Fig. 1C), and was significantly different (p < 0.01) from the control and kale grown with the other media amendments. Hiragonic acid (C16:3n3) is the precursor for the synthesis of C18:3n3, the most abundant n3 PUFA observed in kale. Although we did not conduct any assessment of the levels of desaturase enzymes present in kale, it appears that dry vermicast amended media was effective in enhancing the level or activity of delta 13 desaturase enzyme that catalyzes the biosynthesis of C16:3n3 from C16:2n6^4^. This assumption is based on the fact that no difference was observed in the accumulation of total C18:3n3 in the control and kale grown in the other amended media. Additionally, a concomitant reduction in C16:2n6 was noticed in plants cultivated in dry vermicast media (Fig. 1D). Conversely, only volcanic minerals amended media was effective in enhancing the level of total C18:2n6 fatty acids in kale (Fig. 1D). A clear picture of the differences found among the fatty acid composition of kale grown with different amended media are shown in the principal component analysis in Fig. 1B. The first (F1) and second component (F2) explain 42.9% and 32.7% of the total variance respectively. As it can be observed, due to the highest content in C18:1n9 and C16:3n3, kale grown in dry vermicast media amendments is clearly separated from the other growth media in the first component of the correlation and biplot. The higher N content and electrical conductivity present in vermicast^33^ suggests this amendment had the highest concentrations of ions in solution, which appeared to stimulate the production of C18:1n9 and C16:3n3 fatty acids in kale.

PUFAs (n6 and n3 fatty acids) are essential in humans and need to be obtained from dietary sources such as kale. Diets with a low content of n3 have been attributed as a major factor associated with this global increase in lifestyle related illnesses^6,9^. Kale is a green leafy vegetable with one of the highest concentrations of n3^12^. The findings presented in this work demonstrate that natural amended media, particularly dry vermicast, could be very effective in enhancing the level of n3 fatty acids (C16:3n3) content in kale. This could be a very promising approach to improve the accumulation of the functional components in kale, and the intake of kale enhanced with superior functional lipids (n3 and n9 fatty acids) in human diets.

### Diglycerides as functional lipids in kale

**Figure 2:**
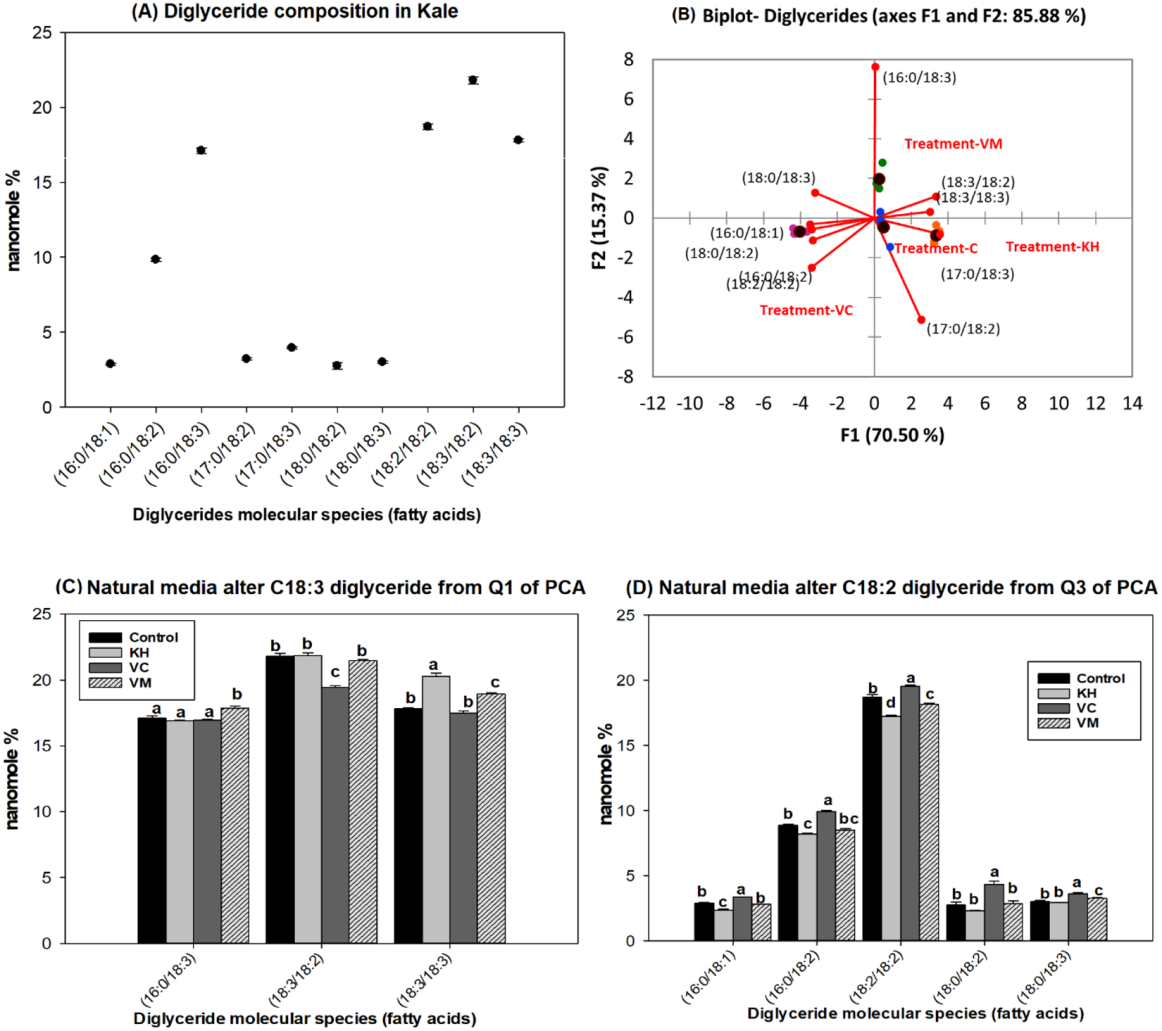
(**A**–**D**). Natural amended media alter the diglycerides (DG) composition of Kale cultivated under control environmental (greenhouse) conditions. (**A**) Observed kale DG, (**B**) Biplot showing relationships between observed kale diglyceride levels and media amendments used for growth. One-way ANOVA showing altered Kale functional lipids segregated in (**C**) quadrant 1 (Q1) and (**D**) Q3 of the correlation circle or biplot following principal component analysis. Values in bar chart (nanomole percent by weight composition) represent means ± standard errors. Means in the same row accompanied by different superscripts are significantly different at LSD α = 0.05, n = 4 per experimental replicate. Control = no media amendment added, KH = potassium humate, VC = dry vermicast, VM = volcanic minerals amendments added to the control media. HESI-MS = heated electrospray ionization mass spectrometry. Ammonium adducts [M + NH_4_]^+^ of DG molecular species were identified using neutral loss of 35 Da in HESI-MS positive mode following lipid class separation using C30 reverse phase chromatography.

Diglycerides (DG) is a relatively new kind of functional lipid present as natural components in various edible plant oils^8,21^. There are several reports in the literature indicating that DG, particularly the 1,3 isoforms have metabolic characteristics distinct from triglycerides. This metabolic characteristic along with structural characteristics appears to produce beneficial effects in regards to the suppression, prevention and/or management of obesity and post prandial lipemia^8^. In addition to triglycerides (not reported), we observed the presence of diglycerides in kale leaves. Regardless of the natural growing media amendment used, the DG molecular species were enriched with C18:2 and C18:3 fatty acids (Fig. 2A–D). As such, the major kale DG molecular species were 16:0/18:3 (17.2 ± 0.02%), 18:2/18:2 (18.70 ± 0.19%), 18:3/18:2 (21.73 ± 0.19%),18:3/18:3 (17.80 ± 0.12%) (Fig. 2A).

Dry vermicast and volcanic mineral amendments were able to modulate the accumulation of DG in kale, while potassium humate was less efficacious (Fig. 2B–D). As such, the media amendments were clearly segregated in distinct quadrants of the biplot (Fig. 2B) based on DG molecular species composition and accumulation. This segregation was based on PCA analysis, where 85.88% of the variability observed in kale DG could be explained by both PC1 and PC2. Dry vermicast seems to be most effective in increasing the overall accumulation of C18:2 DG molecular species in kale (Fig. 2D). Conversely, volcanic minerals enhanced the accumulation of C18:3 molecular species, except for C18:3/C18:2, where no difference was observed compared to the control (Fig. 2B–D).

**Figure 3:**
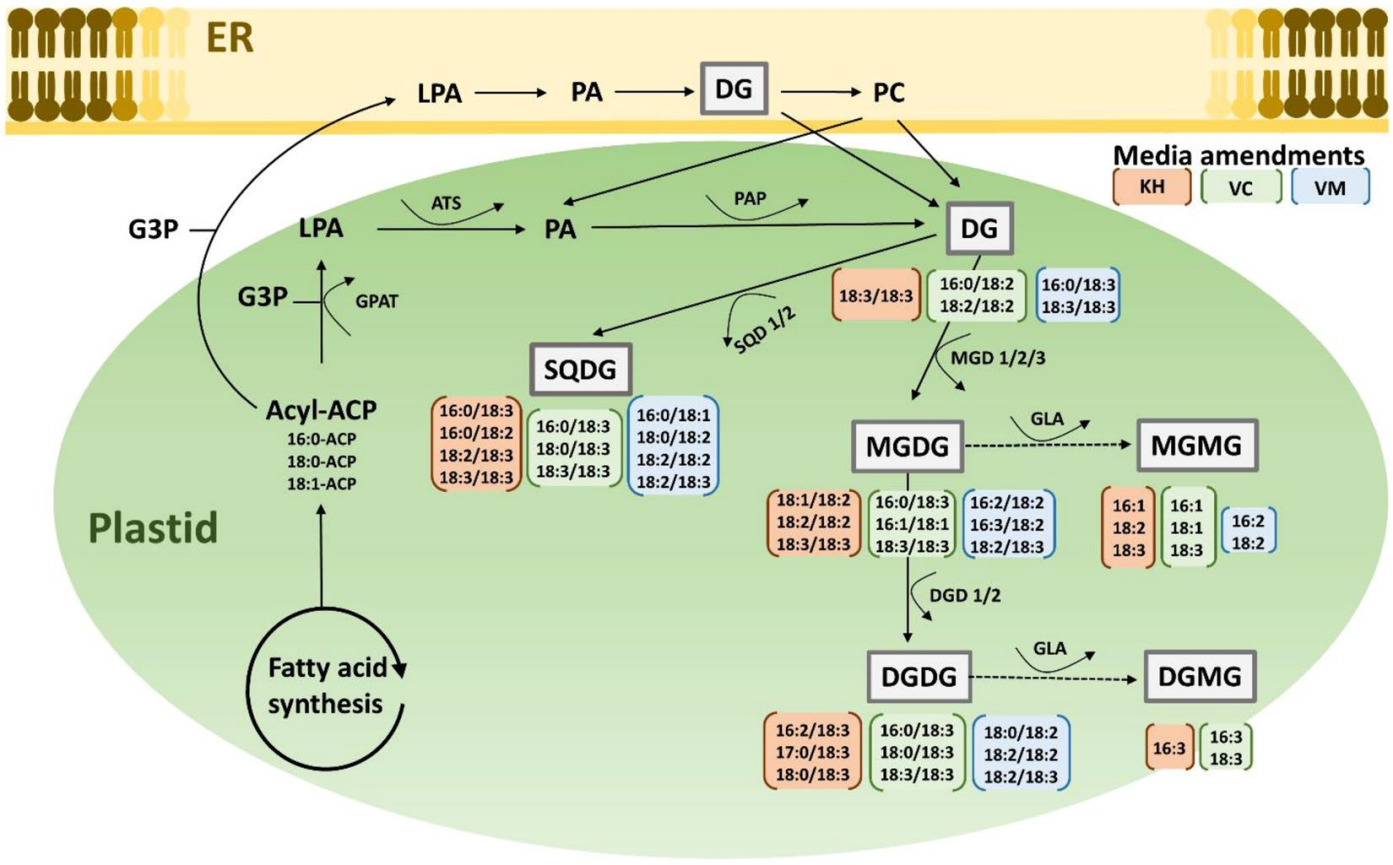
Abbreviated plant biosynthesis glycolipids metabolism. The most abundant molecular species enhanced in the DG and galactolipid classes in kale plants grown in potassium humate (orange) vermicast (green) and volcanic mineral (blue) media amendments are shown. *Abbreviations*: ATS, LPA acyltransferase; DG, diglyceride; DGD, DGDG synthase; G3P, glycerol-3-phosphate; GLA, glycerolypase; GPAT, glycerol-3-phosphate acyl transferase; LPA, lysophosphatidic acid; MGD, MGDG synthase; PA, phosphatidic acid; PAP, phosphatidate phosphatase; SQDG, SQDG synthase; ER, endoplasmic reticulum.

Diglycerides are common in nature, but they tend to exist in very low levels (1–10% w/w)^8,21^. Consequently, their levels are typically enhanced or enriched commercially in food products^8,21^. For example, commercially available DG-rich oils contain approximately 80% DG^21,22^. The findings presented in this work demonstrate the potential of naturally amended media to enhance both the quality and accumulation of diglycerides in kale cultivated under greenhouse conditions. DG are synthesized in both the ER and the chloroplast. In both organelles, glycerol-3-phosphate (G3P) is esterified to form lysophosphatidic acid (LPA), which is transformed into phosphatidic acid (PA) by the lysophosphatidic acid acyltransferase (ATS) enzyme. Phosphatidic acid is then de-phosphorylated by phosphatidate phosphatase (PAP) to form DG^34^ (Fig. 3). Vermicast appears to stimulate the incorporation of C18:2 enriched molecular species in PA and/or the subsequent removal of phosphate (de-phosphorylation) from these PA molecular species leading to the formation of DG molecular species enriched with C18:2 fatty acids. A similar biosynthetic pathway appears to be stimulated by volcanic minerals; except that it is the C18:3 enriched molecular species that are preferentially incorporated into PA or dephosphorylated to produce the higher levels of C18:3 DG molecular species observed in kale cultivated in volcanic minerals (Fig. 3).

Consumption of diglycerides in the diet is low compare to triglycerides^8,21^. The ability of natural amended media to enhance the level and composition of diglycerides in kale present an avenue through which crop producers could produce crops with increase diglycerides. This could be very beneficial in the context of population health, considering the role of diglycerides in suppressing obesity, one of the growing problem worldwide associated with an increased risk for having heart diseases, diabetes mellitus, hypertension and some forms of cancers^21^. The nutritional characteristics and beneficial health effects of diglycerides have been reported in several clinical studies. In these studies, diglycerides were demonstrated to be less likely to be stored as body fat, suppressed body fat accumulation, and lower post prandial serum triglyceride levels^8,21^. Taking these into consideration, it appears the use of natural amended media particularly, dry vermicast and volcanic minerals could be a useful approach to produce kale plants with enhanced levels of diglycerides. Increase access to kale (vegetables) with enhanced diglycerides could aid in increasing consumption of diglycerides in the diet; with potential implications in combating or managing post prandial lipemia and obesity.

### Galactolipids as functional lipids in kale

**Figure 4:**
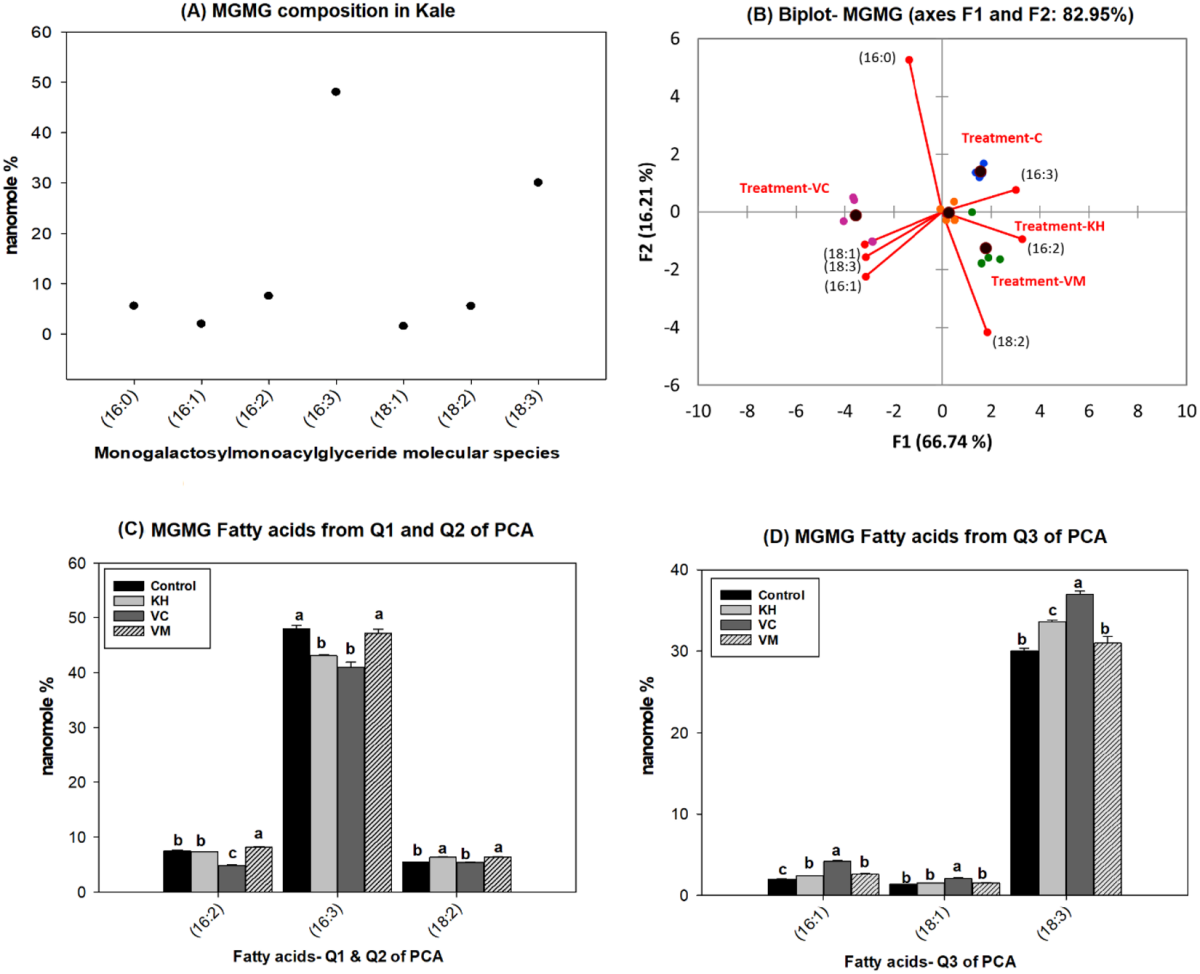
(**A**–**D**). Natural amended media alter the monogalactosylmonoacylglycerides (MGMG) composition of Kale cultivated under control environmental (greenhouse) conditions. (**A**) Observed kale MGMG, (**B**) Biplot showing relationships between observed kale monogalactosylmonoacylglycerides levels and media amendments used for growth. One-way ANOVA showing altered Kale functional lipids segregated in (**C**) quadrants 1 & 2 (Q1 & Q2) and (**D**) Q3 of correlation circle or biplot following principal component analysis. Values in bar chart (nanomole percent by weight composition) represent means ± standard errors. Means in the same row accompanied by different superscripts are significantly different at LSD α = 0.05, n = 4 per experimental replicate. Control = no media amendment added, KH = potassium humate, VC = dry vermicast, VM = volcanic minerals amendments added to the control media.

**Figure 5:**
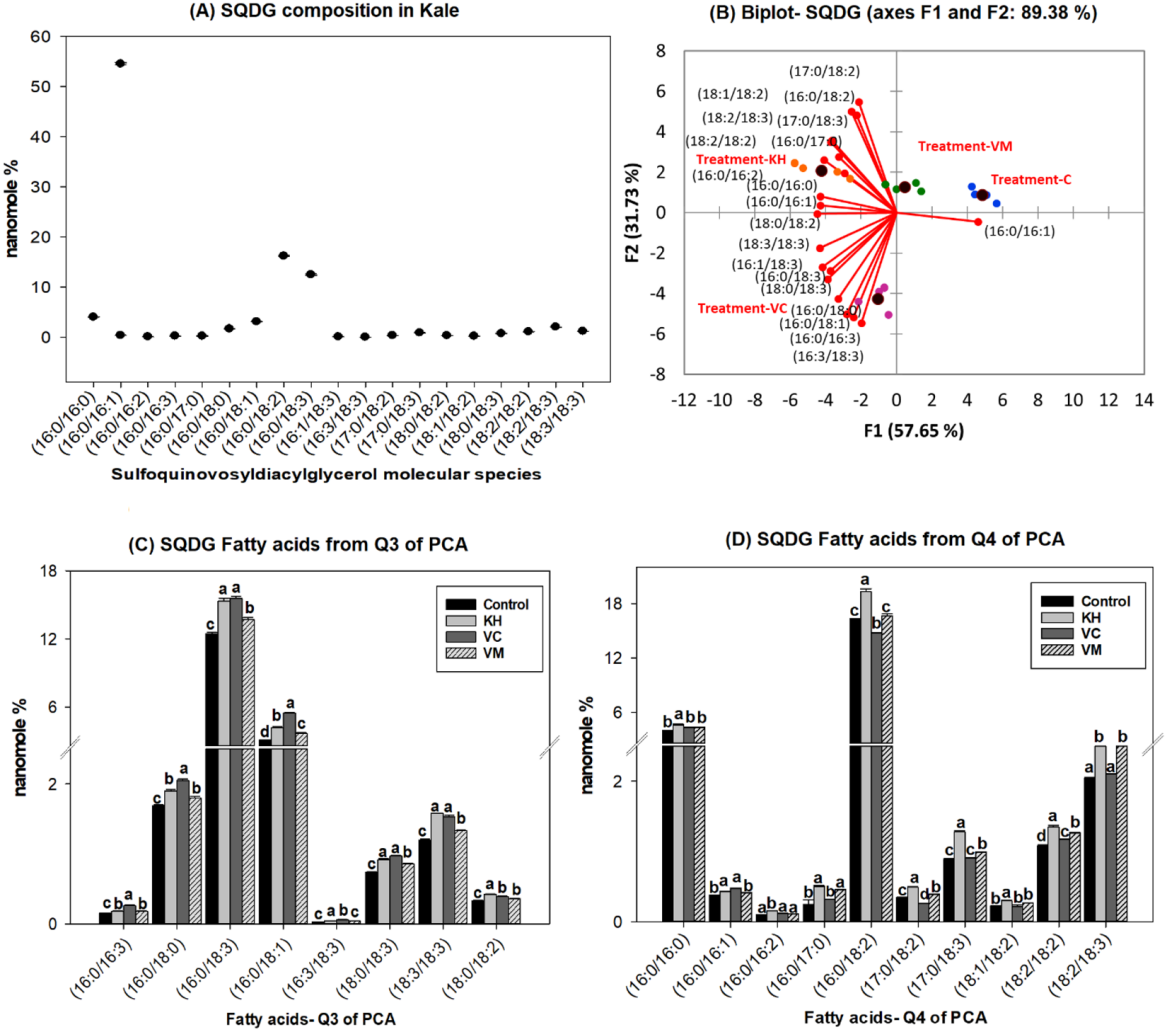
(**A**–**D**). Natural amended media alter the sulfoquinovosyldiacylglycerides (SQDG) composition of Kale cultivated under control environmental (greenhouse) conditions. (**A**) Observed kale SQDG, (**B**) Biplot showing relationships between observed kale sulfoquinovosyldiacylglycerides levels and media amendments used for growth. One-way ANOVA showing altered Kale functional lipids segregated in (**C**) quadrants 3 (Q3) and (**D**) Q4 of correlation circle or biplot following principal component analysis. Values in bar chart (nanomole percent by weight composition) represent means ± standard errors. Means in the same row accompanied by different superscripts are significantly different at LSD α = 0.05, n = 4 per experimental replicate. Control = no media amendment added, KH = potassium humate, VC = dry vermicast, VM = volcanic minerals amendments added to the control media. HESI-MS = heated electrospray ionization mass spectrometry. Deprotonated [M-H]^-^ of SQDG molecular species were identified using a precursor ion scan of *m/z* 225 in HESI-MS negative mode following lipid class separation using C30 reverse phase chromatography.

**Figure 6:**
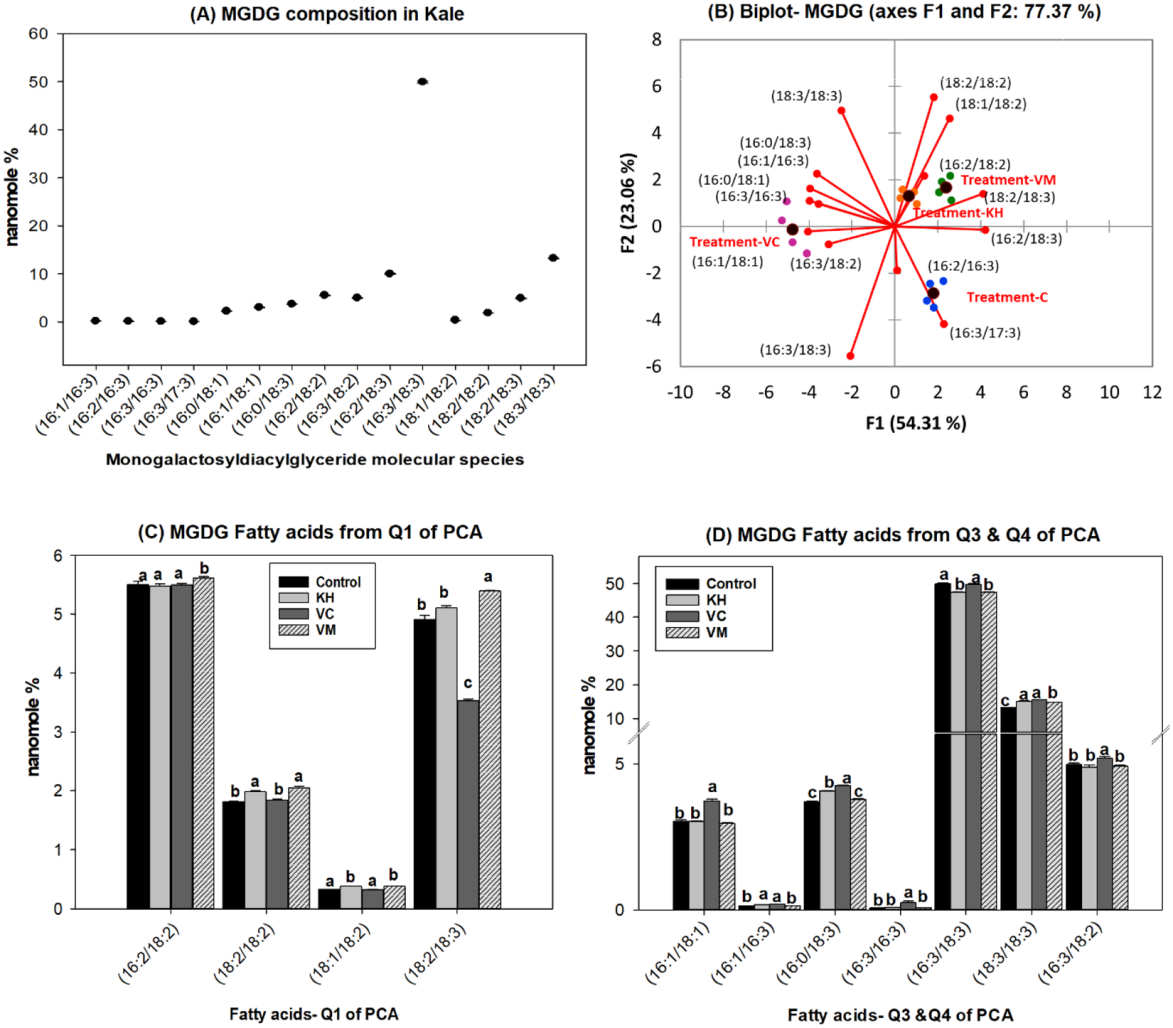
(**A**–**D**). Natural amended media alter the monogalactosyldiacylglycerides composition of Kale cultivated under control environmental (greenhouse) conditions. (**A**) Observed kale monogalactosyldiacylglycerides, (**B**) Biplot showing relationships between observed kale monogalactosyldiacylglycerides levels and media amendments used for growth. One-way ANOVA showing altered Kale functional lipids segregated in (**C**) quadrants 1 (Q1) and (**D**) Q3 & Q4 of correlation circle or biplot following principal component analysis. Values in bar chart (nanomole percent by weight composition) represent means ± standard errors. Means in the same row accompanied by different superscripts are significantly different at LSD α = 0.05, n = 4 per experimental replicate. Control = no media amendment added, KH = potassium humate, VC = dry vermicast, VM = volcanic minerals amendments added to the control media. MGDG = monogalactosyldiacylglycerides. HESI-MS = heated electrospray ionization mass spectrometry.

**Figure 7:**
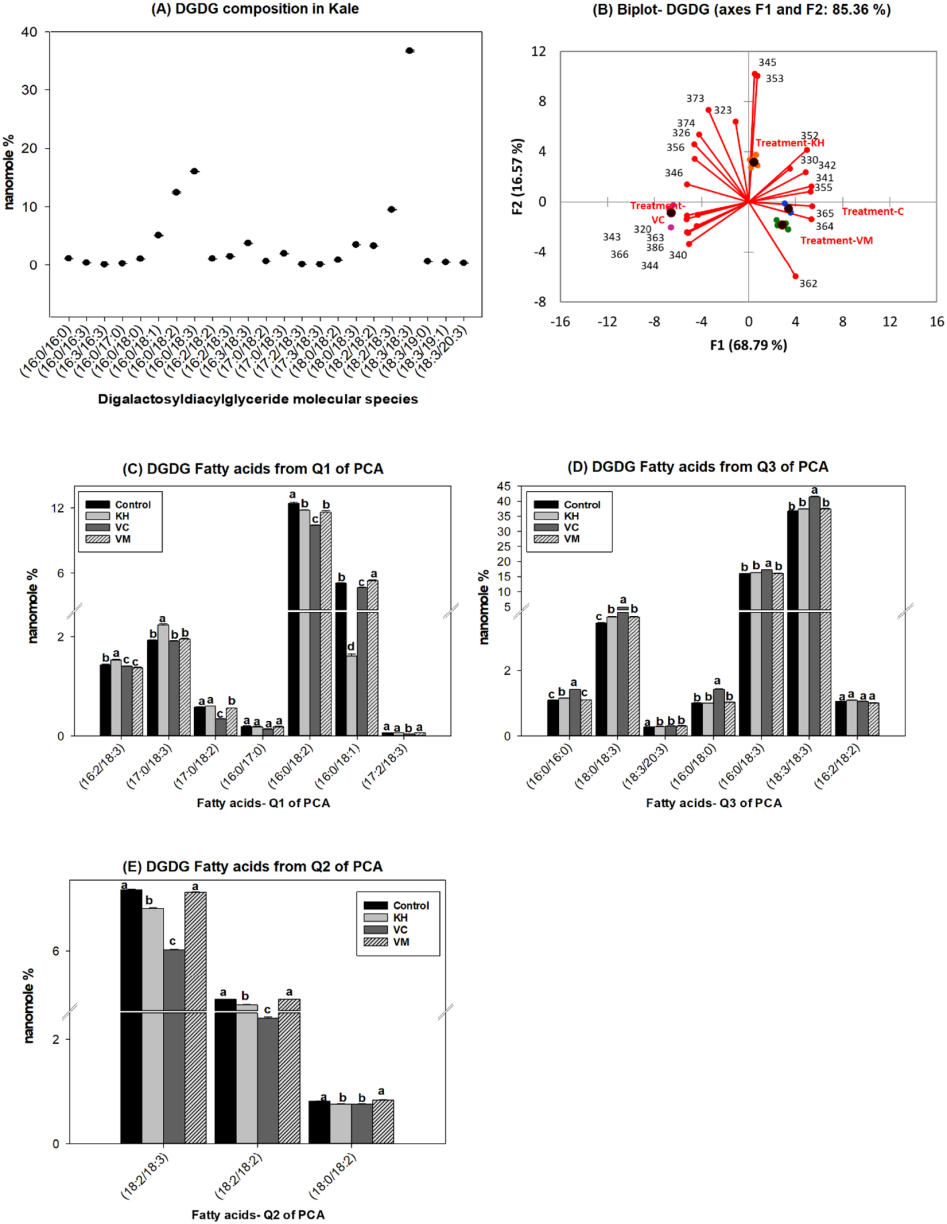
(**A**–**E**). Natural amended media alter the digalactosyldiacylglycerides composition of Kale cultivated under control environmental (greenhouse) conditions. (**A**) Observed kale digalactosyldiacylglycerides, (**B**) Biplot showing relationships between observed kale digalactosyldiacylglycerides levels and media amendments used for growth. One-way ANOVA showing altered Kale functional lipids segregated in (**C**) quadrants (Q1), (**D**) Q3 (**E**) Q2 of correlation circle or biplot following principal component analysis. Values in bar chart (nanomole percent by weight composition) represent means ± standard errors. Means in the same row accompanied by different superscripts are significantly different at LSD α = 0.05, n = 4 per experimental replicate. Control = no media amendment added, KH = potassium humate, VC = dry vermicast, VM = volcanic minerals amendments added to the control media. DGDG = digalactosyldiacylglycerides. HESI-MS = heated electrospray ionization mass spectrometry. Formic acid adducts [M + HCOO]− of DGDG molecular species were identified using a precursor ion scan of *m/z* 397, 415 in HESI-MS negative mode following lipid class separation using C30 reverse phase chromatography. Diacyl species were used for the correlation circle, scope and biplot plots because use of the molecular species as in previous figures would make these figures unreadable. Please see bar chart for the corresponding figures as separated per quadrant in the score, biplots and correlation circle. DGDG molecular were identified using a characteristic fragment ion of *m/z* 397, 415 in HESI-MS negative mode following lipid class separation using C30 reverse phase chromatography.

Dark green leafy vegetables, such as kale, are abundant in galactolipids^6,27^. The most abundant galactolipids present are SQDG, MGDG and DGDG. Consistent with this observation, SQDG (Fig. 5), MGDG (Fig. 6) and DGDG (Fig. 7) were observed as the major galactolipids in kale accompanied by MGMG (Fig. 4) and DGMG (see Supplementary Fig. S1) as minor components. DG is a central intermediate in galactolipids synthesis, being the precursors for the formation of MGDG. Galactose is transferred from uridine-5′-diphosphate galactose (UDP-galactose) to DG by MGDG synthase (MGD) to form MGDG in the chloroplast. MGDG then become the substrate used by DGDG synthase (DGD) to synthesize DGDG^35^. MGMG and DGMG are lysogalactolipids formed by the hydrolysis of MGDG and DGDG (Fig. 3). Galactolipids are known to be important sources of n3 and n6 fatty acids in leafy vegetables. For example, from the total MGDG, up to 90% was observed to be C18:3n3 in stem and leaf vegetables^6^. Similarly, we observed that kale galactolipids were enriched with C16:3, C18:2, and C18:3 fatty acids (Figs 4–7, see Supplementary Fig. S1). As such, the following were the predominant molecular species present within each galactolipid class observed in kale: C16:3 (48.01 ± 0.55%) and C18:3 (30.4 ± 0.36%) in MGMG (Fig. 4A); C16:0/C16:1 (54.52 ± 0.27%) and 16:0/18:2 (16.23 ± 0.11%) in SQDG (Fig. 5A); 16:3/18:3 (49.94 ± 0.2%) and 18:3/18:3 (13.94 ± 0.9%) in MGDG (Fig. 6A); C18:3 (70.87 ± 0.17%) in DGMG (see Supplementary Fig. S1A); 16:0/18:3 (16.0 ± 0.07%) and 18:3/18:3 (36.74 ± 0.18%) in DGDG (Fig. 7A). Other molecular species containing C16:3, C18:3 and C18:2 fatty acids were also observed to exist in minor quantities in every class of kale galactolipids (Figs 4–7, see Supplementary Fig. S1). Cumulatively, their contributions to the total polyunsaturated fatty acid content in kale galactolipids were observed to be over 85%, except in SQDG (Fig. 5A); where C16:1 fatty acid enriched molecular species predominated and accounted for over 60%.

Galactolipids are a subclass of glycolipids, which are the major membrane lipids present in the chloroplast of green plants^6,27^. Several studies have demonstrated the bioactivities of galactolipids from vegetables as important nutraceuticals with documented effects on the treatment or prevention of cancer and anti-inflammatory diseases such as arthritis^6,16,36^. Consequently, there are several recent patents granted in relation to the application of galactolipids in functional foods, plant medicines, and dietary supplements for the prevention of these diseases^6,17–20^. The evidences in these patents, as well as findings reported in the literature, suggested that galactolipids in vegetables play an important role as functional ingredients; and that their bioactivities are associated in part with the significant correlation observed between high vegetable consumption and reduced risks for developing cancer, anti-inflammatory and cardiovascular diseases^3,6,7,36,37^. Thus, production of vegetables with enhanced levels of galactolipids could be a useful strategy to improve access and consumption of higher levels of these health promotive functional compounds in the diet. Modulation of the growth environment has been shown to significantly influence the accumulation of galactolipids in vegetables^10^. In the current study, we attempted to use natural amended media to alter the levels of galactolipids as functional lipids in kale cultivated under controlled environmental conditions. In all galactolipids classes evaluated, over 77% of the variability could be explained by PC1 and PC2 as depicted by the outputs following PCA (Figs 4–7, see Supplementary Fig. S1). PCA analysis was very effective in grouping the media amendments based on similarities or differences in altering galactolipids molecular species within each galactolipids class.

Overall, all the three media amendments were effective in altering the levels of kale galactolipids molecular species (Figs 4–7, see Supplementary Fig. S1). In all cases, kale plants cultivated in the potassium humate and the dry vermicast amended media were segregated in different quadrants of the biplots (Figs 4–7, see Supplementary Fig. S1) compared to the control plants. As a general trend, dry vermicast significantly (p < 0.01) promoted the accumulation of C18:3 enriched molecular species, while simultaneously either reducing or not altering the accumulation of C18:2 enriched galactolipid molecular species. In the instances where a reduction in C18:3 enriched molecular species were observed (i.e. DGMG and MGMG), a concomitant increase in C16:3 enriched molecular species, were also observed or *vice versa* (Fig. 4, see Supplementary Fig. S1). These findings suggested that dry vermicast amended media modulate the contents of galactolipids molecular species in kale by suppressing C18:2 fatty acid syntheses, while simultaneously enhancing the level of either C16:3 or C18:3 enriched molecular species. Potassium humate on the other hand, was observed to simultaneously enhanced the accumulation of both C18:2 and C18:3 fatty acids enriched molecular species (Figs 4–6C–D), except in DGMG (see Supplementary Fig. S1E) and DGDG (Fig. 7C–E). In addition, the accumulation of monounsaturated fatty acids (C16:1 and C18:1) enriched galactolipids molecular species were observed to increase in kale grown in both potassium humate and dry vermicast treatments (Figs 4D, 5C,D and 6D).

This is, potassium humate was observed to be effective in modulating the levels of C18:2, C18:3 and C18:1 galactolipid molecular species in kale. In most instances, this modulation was an overall increase accumulation of these molecular species. Volcanic minerals on the other hand, were observed to be the least effective in altering kale galactolipids (Figs 4–7, see Supplementary Fig. S1). Following PCA analysis, volcanic minerals and the control treated plants clustered in the same quadrants of the score and biplots based on their accumulation of galactolipids. Only in MGDG, plants cultivated in media amended with volcanic minerals were not clustered with the control treated plants (Fig. 6B–D). Here we observed volcanic minerals enhanced the accumulation of C18:2 fatty acid enriched MGDG molecular species (Fig. 6B,C). It is also interesting to note that the production of kale in volcanic minerals amended media was very effective in altering the level of SQDG molecular species (Fig. 5C,D). This media amendment increased the accumulation of C18:3 and C18:2 enriched SQDG molecular species in kale (Fig. 5). This effect was also observed in plants grown with dry vermicast and potassium humate treatments. The most abundant molecular species enhanced in the galactolipid classes in kale plants grown in potassium humate, vermicast and volcanic mineral amendments are shown in Fig. 3. Collectively, these findings suggest all three natural growing media amendments can be used to enrich the levels of galactolipids in kale plants grown under controlled environment conditions. Dry vermicast appears to be better suited for the enhancement of C16:3 and C18:3 (n3) enriched molecular species in kale (Fig. 3). Potassium humate appears to be more effective in enhancing the C18:3 levels in SQDG and MGDG molecular species (Fig. 3). These results suggest potassium humate seems to stimulate the activities of the glycosyltransferases (SQDG and MGDG synthase) that catalyze the transfer of the sulfoquinovose and monogalactose moieties respectively to DG species containing C18:3 fatty acids. This action possibly accounted for the increase formation of C18:3 enriched SQDG and MGDG molecular species observed in kale grown in potassium humate amended media (Fig. 3). Like potassium humate, both volcanic minerals and dry vermicast appears to also stimulate glycosyltransferase activities during glycolipid synthesis in kale. However, there seems to be a preference for dry vermicast to catalyze the transfer of galactose or sulfoquinovose moieties to C18:3 enriched DG, MGDG or SQDG molecular species; thus accounting for the higher levels of C18:3 enriched molecular species in these classes of galactolipids following cultivation in dry vermicast amended media (Fig. 3). Conversely, volcanic minerals appeared to be less discriminate in catalyzing the transfer of galactose or sulfoquinovose moieties to C18:1, C18:2 or C18:3 enriched molecular species across all lipid classes of galactolipids. This mode of action may account for the increase levels of C18:1, C18:2 or C18:3 enriched molecular species observed in the plants grown with volcanic mineral amendment (Fig. 3).

This discovery is very promising in the context of producing vegetable crops under control environmental conditions with enhanced levels of galactolipids as functional ingredients. This is of relevance considering several recent patents being granted for the potential use of the health promotive properties of galactolipids to produce medicinal and functional food products for the prevention or treatment of anti-inflammatory illnesses and cancer^6,17–20^.

### Phytosterols as functional lipids in kale

Beta-sitosterols were the only phytosterol observed in kale regardless of the media amendment used for cultivation. Four fatty acids (C18:2, C18:3, C28:6, and C38:5) were observed to be esterified to beta-sitosterol in kale (see Supplementary Fig. S2). The scientific literature is replete with evidence of phytosterols including beta-sitosterol in decreasing plasma LDL and cholesterol levels; as well as reducing the risk for developing cardiovascular diseases^8^. The results obtained indicated that natural growing media amendments had very marginal overall effects in altering kale beta-sitosterols under the experimental conditions used in this study (see Supplementary Fig. S2).

## Conclusions

The results obtained demonstrate that natural growth media amendments are very effective in modulating the levels of functional lipids (i.e. PUFAs, diglycerides and galactolipids) in kale cultivated under control environmental conditions. Dry vermicast and volcanic mineral amended media appeared to be the most effective. This could be a useful strategy for the production of functional foods in control environment production systems, such as greenhouses or environmental growth rooms. It is universally accepted that population health is influenced by diet, and that there are strong relationships between diets high in vegetables, and reduce risks of developing common lifestyle related illnesses. Considering that the diet together with the lifestyle habits are important risk factors related with the development of certain illnesses (coronary diseases, type 2 diabetes, cancer, osteoporosis and obesity, this work suggests the use of natural growing media amendments, particularly, dry vermicast and volcanic minerals could be a useful approach to produce kale plants with enhanced levels of functional lipids. Increase access to kale (vegetables) with enhanced functional lipids could aid in increase consumption of these health promotive compounds in the diet, with potential implications in reducing the risk factors associated with developing common lifestyle related illnesses.

## Materials and Methods

### Chemicals

The following chemicals were purchased from Fisher Scientific (Ottawa, ON, Canada): LC-grade chloroform, methanol, acetonitrile, formic acid, acetic acid and ammonium acetate. For solutions preparation, deionized water (PURELAB Purification System, ELGA Labwater, ON, Canada) was employed. Phospholipids, sphingolipids and glycolipids standards were used (Avanti Polar Lipids Inc., Alabaster, AL, USA).

### Kale greenhouse production

Seed of Organic Kale ‘*Ripbor*’ (Bejo Zaden b.v., Warmenhuizen, The Netherlands) were planted in trays and transferred into plastic pots with 25.4 cm-diameter containing 1 kg of moist Pro-mix BX™ potting medium (Premier Horticulture Inc., Quakertown, PA, USA). The composition of Pro-mix BX™ was 75–85% sphagnum peat moss, vermiculite, wetting agent, dolomitic, horticultural grade perlite and calcitic limestone. Mycorrhizal fungus, *Glomus intraradices* was used to pre-inoculate all the media. Kale growth conditions in the greenhouse were as follows: 24 °C and 16 °C at daytime and night respectively with a 71% average humidity. High pressure sodium lamps (600 W HS2000) with NAH600.579 ballast (P.L. Light Systems, Beamsville, ON, Canada) was used as supplementary lighting. Plants were irrigated every 2 days with 200 ml of tap water until harvesting.

### Experimental treatments

Three different natural and organic growing media amendments were employed in this study: potassium humate (KH), dry vermicasts (VC) and volcanic minerals (VM). The natural media amendments were individually added to the pots with Pro-mix BX^TM^ as described by the manufacturer. This is: 47.5 g KH/pot, 50 g VC/pot, and 100 g VM/pot. As a control (C) treatment, a pot containing only Pro-mix BX™ was used^29^. Seedlings were transplanted in the pots containing the natural media amendments or Pro-Mix (control). Weekly rearrangements of the pots were performed to mitigate variations in the greenhouse microclimate employing a randomized block experimental design. No synthetic chemicals or fertilizers were added to the medium or the plants in this experiment. Ten replications (n = 10 plants) were employed per experimental treatment (C, KH, VC, VM).

### Extraction of Kale lipids

One gram (1 g) of leave was collected from each plant replication (n = 10) and pooled. Pooled leaves from each treatment (C, KH, VC, VM) were incubated in hot isopropanol and subsequently frozen in liquid nitrogen and cryo-homogenized to obtain kale powder. The kale powder was used for lipids extraction^33^. Briefly, 25 mg of powder was dissolved in methanol/chloroform/0.01% butylated hydroxytoluene (1:2:0.0003; v/v/w with a 10:1 (w/v) proportion). The sample mixture was centrifuged (10,000 × g −15 min) and the supernatant collected. One milliliter (1 mL) of potassium chloride (0.25%) was added to the supernatant and the mixture incubated (70 °C) for 10 min. After allowing samples to cool down, the lower phase (organic phase containing the lipids) was taken, dried under nitrogen and the amount of lipid recovered determined gravimetrically^38,39^. The lipids were then re-suspended in chloroform: methanol (2:1 v/v), and the functional lipids analyzed using either gas chromatography-mass spectrometry/flame ionization detection (GC-MS/FID), or ultra-high performance liquid chromatography coupled to heated electrospray ionization tandem high resolution mass spectrometry (UHPLC-HESI-HRMS/MS).

### Analysis of kale fatty acids using GC/MS and GC/FID

Kale fatty acids were converted to fatty acids methyl esters (FAMEs) as follows: To 300 µL of the extracted lipids, 50 µL of C19:0 FA (1 mg/mL in chloroform:methanol (2:1 v/v)) was added as internal standards, and the samples dried under nitrogen. The fatty acids in the samples were methylated by adding 100 μL of methanolic-HCl 3 N (Sigma-Aldrich, ON, Canada), followed by vortexing and incubation (80 °C) for 30 min. Following incubation, distilled water (0.8 mL) was added to the cooled samples, and the FAMEs extracted with 500 μL of hexane aliquots (x3). The fractions were combined, dried (under N_2_) to zero and re-suspended in hexane (50 μL). The FAMEs obtained were analyzed using GC/MS and GC/FID.

*GC/MS analysis of kale FAMEs* was done using a Trace 1300 gas chromatography (GC) coupled to a TSQ 8000 Triple Quadrupole mass spectrometer (ThermoScientific, Brampton, ON, Canada). Methylated fatty acids in the sample were separated using a DB23 high resolution column (60 m × 0.25 mm × 0.25 μm) (Agilent Technologies, Santa Clara, CA, USA) using He as the carrier gas (flow rate:1 ml/min). The sample was injected (1 μl) in the instrument using a Tri-plus auto-sampler. The operation conditions of the instrument were as follows: split mode injection (20:1), oven temperature programed initially at 80 °C (held for 5 min), then increased at 4 °C/min until 220 °C (held for 5 min), finally ramped to 240 °C at 4 °C/min (held for 10 min). The identification of the methylated fatty acids was done by mass spectrum elucidation and retention times and mass spectra comparison with those of the commercial standards (Supelco FAME mix C8–C24, Supelco 37 component mix, Supelco PUFA No. 3; Sigma Aldrich, ON, Canada). Standard curves were employed to determine the amount of individual fatty acids. Values are presented on a nmole % basis.

*GC/FID analysis* of kale fatty acids was conducted using a Trace 1300 gas chromatography coupled to a Flame Ionization Detector (Thermo Fisher Scientific, Waltham, MA, USA). FAMEs were separated in a DB-23 column (30 m × 0.25 mm × 0.25 μm; Agilent Technologies, Santa Clara, CA, USA) using He as the carrier gas (flow rate: 1 ml/min). The sample was injected (1 μl) in the instrument using a Tri-plus auto-sampler. The operation conditions were as follows: Injection system was splitless mode. The oven temperature was set up at 50 °C (held for 1 min) and increased 20 °C/min to 175 °C (held for 1 min), afterwards, it was increased again to 230 °C at 4 °C/min (held for 5 min). FAMEs were identified by comparison of the retention times with those of the standards (Supelco PUFA No. 3 mix, Supelco 37 component mix, Supelco FAME mix C8–C24; Sigma Aldrich, ON, Canada). C19:0 FAME was employed as internal standard. Standard curves were employed to determine the amount of individual fatty acids, and values are presented as nmole %.

### Analysis of kale intact functional lipids using UHPLC-C30RP-HESI-HRMS/MS

The functional lipids classes of kale, as well as their molecular species, were separated by means of ultra-high performance liquid chromatograph coupled to C30 reverse phase chromatography and heated electrospray ionization high resolution tandem mass spectrometry (UHPLC-C30RP-HESI- HRMS/MS). A Q-Exactive Orbitrap mass spectrometer (Thermo Scientific, MO, USA) coupled with an automated Dionex UltiMate 3000 UHPLC system controlled by Chromeleon software was used for the lipid analysis. Lipids were separated on an Accucore C30 column (150 × 2 mm I.D., particle size: 2.6 µm, pore diameter: 150 Å; ThermoFisher Scientific,ON, Canada). The solvent system used was: acetonitrile: H_2_O (60:40 v/v), 10 mM ammonium formate and formic acid (0.1%) as solvent A; isopropanol:acetonitrile:water (90:10:1 v/v/v), 10 mM ammonium formate and formic acid (0.1%) as solvent B.

UHPLC-C30RP separation was done by injecting the complex lipid mixture (10 µL) dissolved in chloroform:methanol (2:1 v/v). The column oven temperature was 30 °C and the flow rate 0.2 mL/min. The following system gradient was used: Solvent B: 30% for 3 min; increased to 43% over 5 min, 50% in 1 min, 90% over 9 min, 99% over 8 min and kept it there for 4 min. The column was re-equilibrated to 70% of solvent A (starting conditions) for 5 min prior to the next injection. Full scan HESI-MS and MS/MS acquisitions were performed in negative and/or positive modes on the Q-Exactive Orbitrap mass spectrometer, and the instrument controlled by X-Calibur software 4.0. Parameters used in the Orbitrap mass spectrometer were: sheath gas 40, auxiliary gas 2, 3.2 kV ion spray voltage, 300^o^C capillary temperature; 30 V S-lens RF; mass range between 200–2000 m/z; full scan mode at a resolution of 70,000 m/z; top-20 data dependent MS/MS at a resolution of 35,000 m/z and collision energy of 35 (arbitrary unit); 35 min injection time for C30RP chromatography; 1 m/z isolation window; automatic gain control target: 1e5 with dynamic exclusion setting of 5.0 s. Calibration of the instrument was periodically done to 1 ppm using ESI negative and positive external calibration solutions (ThermoScientific, MO, USA). Negative and positive ion modes tune parameters were optimized using PC (18:1(9Z)/18:1(9Z), Cer d18:1/18:1(9Z), PG (18:1 (9Z)/18:1(9Z), SQDG 18:3(9Z,12Z,15Z)/16:0, MGDG 18:3(9Z,12Z,15Z)/16:3(7Z,10Z,13Z) and DGDG 18:3(9Z,12Z,15Z)/18:3(9Z,12Z,15Z) standards (Avanti Polar Lipids, Alabama, USA).

### Data processing (UHPLC-C30RP-HESI-HRMS/MS)

Data was acquired and processed using X-Calibur 4.0. (ThermoScientific, MO, USA) and LipidSearch version 4.1 (Mitsui Knowledge Industry, Tokyo, Japan) software packages. For the identification and semi-quantification of the lipid classes and their molecular species present in the complex lipid samples, LipidSearch was used. The parameters used for identification were as follow: Target database: Q-Exactive; precursor tolerance: 5 ppm; product tolerance: 5 ppm; product ion threshold: 5%; m-score threshold: 2; Quan m/z tolerance: ±5 ppm; Quan RT (retention time) range: ± 1 min; use of all isomer filter and ID quality filters A, B, and C; Adduct ions: [M + H]^+^ and [M + NH_4_]^+^ for positive ion mode, and [M-H]^−^, [M + HCOO]^−^, and [M-2H]^2−^ for negative ion mode. The functional lipid classes selected for the search were: DG (diacylglycerol), SiE (sitosterol), StE (stigmasterol), AGlcSiE (acylated glucosyl-β-sitosterol), MGMG (monogalactosylmonoacylglycerol), DGMG (Digalactosylmonoacylglycerol), MGDG (monogalactosyldiacylglycerol), DGDG (digalactosyldiacylglycerol), and SQDG (sulfoquinovosyldiacylglycerol). Following identification, the observed lipid classes and molecular species were aligned and merged using the following alignment parameters: search type: product; experiment type: LC-MS; alignment method: mean; RT tolerance: 0.15; calculate unassigned peak area: on; filter type: new filter; top rank filter: on; main node filter: all isomer peaks, M-score threshold: 5; ID quality filter: A,B,C. Lipid standards were employed for the optimization of the alignment parameters prior to untargeted lipidomics analysis. Fatty acyl chains position identification present in the molecular species found in the lipid classes of each sample was based on the fragmentation patterns of the MS/MS spectra, and manually confirmed using X-Calibur 4.0 according to the rules established for tandem mass spectrometry^40–42^.

### Statistical Analysis

A supervised multivariate analysis approach using XLSTAT (Addinsoft, New York, USA) was applied to the kale functional lipid classes identified by Lipid Search and Xcalibur softwares. Principal component analysis (PCA) was employed to determine which group of kale functional lipids could segregate the natural media amendments based on similarities or differences based on the lipid profiles into different quadrants of the correlation circle and biplot. The effects of the treatments (natural media amendments) on the variation in levels of functional lipids that segregated the treatments based on similarities or differences in the correlation circle or biplot was determined by one-way analysis of variance (ANOVA). The means of those treatments which effect was significant were compared by Fisher’s Least Significant Difference (LSD, α = 0.05) analysis. A total of ten plants (pooled) were used per treatment and each treatment replicated four times. Figures were prepared using XLSTAT and SigmaPlot 13.0 software programs (Systat Software Inc., San Jose, CA).

## Electronic supplementary material

Figure S1 ans S2
