# Supplementary material for: The use of natural media amendments to produce kale enhanced with functional lipids in controlled environment production system
Source: Sci Rep. 2018 Oct 3;8:14771. doi: 10.1038/s41598-018-32866-5 (PMC6170470; doi:10.1038/s41598-018-32866-5)
Supplement: Supplementary file 1 — Figure S1 ans S2 [file 41598_2018_32866_MOESM1_ESM.pdf]

The use of natural media amendments to produce kale enhanced with functional lipids in control environment production system

Natalia P. Vidal[a*], Huong T. Pham[a], Charles Manful[a], Ryley Pumphrey[a], Muhammad Nadeem[a,c], Mumtaz Cheema[a], Lakshman Galagedara[a], Adedayo Leke-Aladekoba[b], Lord Abbey[b], Raymond Thomas[a*]

[a]School of Science and the Environment/ Boreal Ecosystem Research Initiative, Grenfell Campus, Memorial University of Newfoundland, Corner Brook, A2H 5G4, Canada

[b]Department of Plant, Food, and Environmental Sciences, Dalhousie University, Truro, NS, B2N 5E3, Canada

[c]Department of Environmental Sciences, COMSATS Institute of Information Technology, Vehari 61100, Pakistan

Corresponding authors:

* rthomas@grenfell.mun.ca , phone: +1 709 637 7161

* nprietovidal@grenfell.mun.ca phone: +1 709 639 4676

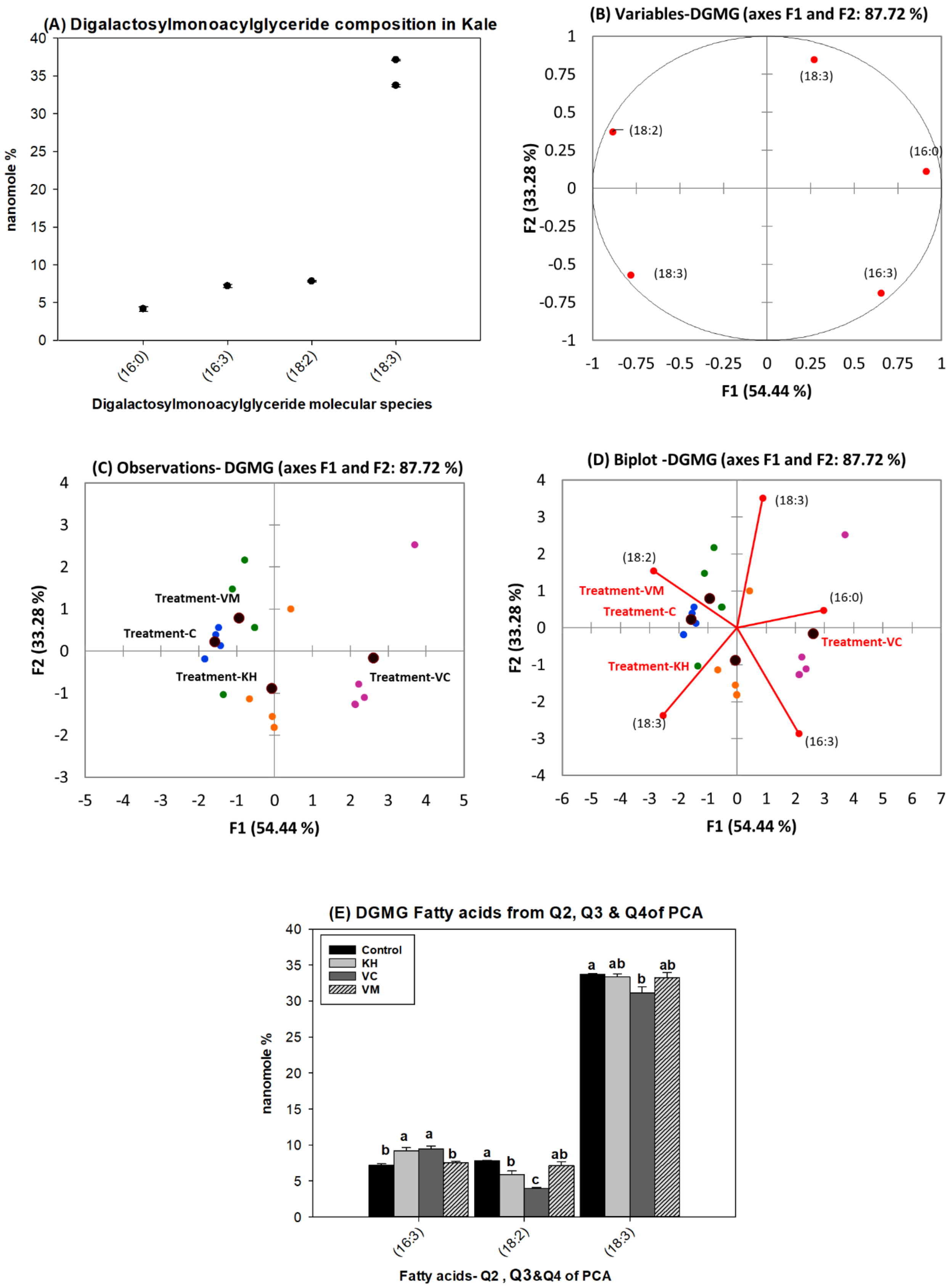
(A) Digalactosylmonoacylglyceride composition in Kale
nanomole %
Digalactosylmonoacylglyceride molecular species
(16:0)
(16:3)
(18:2)
(18:3)
(B) Variables-DGMG (axes F1 and F2: 87.72 %)
F1 (54.44 %)
F2 (33.28 %)
(C) Observations- DGMG (axes F1 and F2: 87.72 %)
Treatment-VM
Treatment-C
Treatment-KH
Treatment-VC
(D) Biplot -DGMG (axes F1 and F2: 87.72 %)
(E) DGMG Fatty acids from Q2, Q3 & Q4of PCA
Control
KH
VC
VM
Fatty acids- Q2 , Q3&Q4 of PCA

Figure S1A-E. Natural amended media alter the digalactosylmonoacylglycerides composition of Kale cultivated under control environmental (greenhouse) conditions. (A) Observed kale digalactosylmonoacylglycerides, **(B)** Correlation circle showing relationships between observed kale digalactosylmonoacylglycerides and location on axis F1 and F2. **(C),** Biplot showing relationships between observed kale digalactosylmonoacylglycerides levels and media amendments used for growth. **(D)** Observation principal components showing segregation of media amendments on axis F1 and F2 based on kale digalactosylmonoacylglycerides composition. (E) One-way ANOVA showing altered Kale functional lipids segregated in **(E)** all four quadrants (Q1, 2, 3 &4) of correlation circle or biplot following principal component analysis. Values in bar chart (nanomole percent by weight composition) represent means ± standard errors. Means in the same row accompanied by different superscripts are significantly different between genotypes at LSD = 0.05, n = 4 per experimental replicate. Control = no media amendment added, KH = potassium humate, VC = vermicompost, VM = volcanic minerals amendments added to the control media. DGMG = monogalactosyldiacylglycerides. HESI-MS = heated electrospray ionization mass spectrometry. Formic acid adducts $[M+HCOO]^-$ of DGMG molecular species were identified using a precursor ion scan of *m/z* 397, 415 in HESI-MS negative mode following lipid class separation using C30 reverse phase chromatography.

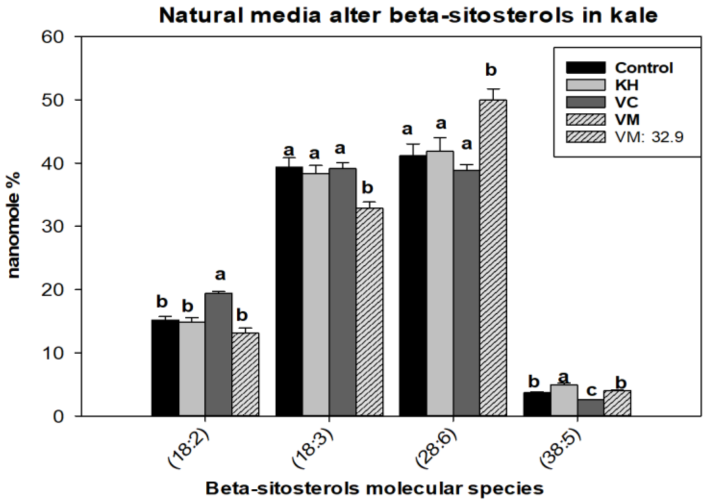

Figure S2. Natural amended media alter the beta-sitosterol composition of Kale cultivated under control environmental (greenhouse) conditions. One-way ANOVA showing altered Kale functional lipids. Values in bar chart (nanomole percent by weight composition) represent means ± standard errors. Means in the same row accompanied by different superscripts are significantly different between genotypes at LSD = 0.05, n = 4 per experimental replicate. Control = no media amendment added, KH = potassium humate, VC = vermicompost, VM = volcanic minerals amendments added to the control media. HESI-MS = heated electrospray ionization mass spectrometry. Ammonium adducts $[M+NH_4]^+$ of beta sitosterol molecular species were identified using a precursor ion scan of *m/z* 397 in HESI-MS positive mode following lipid class separation using C30 reverse phase chromatography.
